# Could METS-VF provide a clue as to the formation of kidney stones?

**DOI:** 10.3389/fendo.2023.1166922

**Published:** 2023-05-22

**Authors:** Zhenyu Guo, Guoxiang Li, Yan Chen, Shuai Fan, Shuai Sun, Yunwu Hao, Wei Wang

**Affiliations:** ^1^ Department of Urology, the First Affiliated Hospital of Anhui Medical University, Hefei, China; ^2^ Institute of Urology, Anhui Medical University, Hefei, China; ^3^ Anhui Province Key Laboratory of Genitourinary Diseases, Anhui Medical University, Hefei, China; ^4^ Department of General Practice, Wuhu City Second People`s Hospital, Wuhu, China; ^5^ Department of Urology, Lu’an Hospital Affiliated of Anhui Medical University, Lu’an, China

**Keywords:** METS-VF, kidney stones, obesity, NHANES, clue

## Abstract

**Objective:**

The lifetime occurrence rate of kidney stones is 14%, making it one of the most prevalent urological conditions. Other contributing elements, such as obesity, diabetes, diet, and heredity, are also taken into account. Our research sought to explore the potential link between high visceral fat scores (METS-VF) and the occurrence of kidney stones, as a means of understanding how to prevent them.

**Methods:**

This research utilized data from the National Health and Nutrition Examination Survey (NHANES), mirroring the demographics of the United States. We carried out an in-depth analysis of the connection between METS-VF and kidney stones, based on data from 29,246 participants in the National Health and Nutrition Examination Survey spanning 2007 to 2018, involving logistic regression, segmentation, and dose-response curve analysis.

**Results:**

Our study of 29,246 potential participants found that METS-VF was positively associated with the prevalence and progression of kidney stones. After subgroup analysis by gender, race, blood pressure, and blood glucose, our results showed that the ORs for METS-VF and kidney stones were (1.49, 1.44) in males and females, respectively; while in Mexicans, whites, blacks, and In other populations, the OR values were (1.33, 1.43, 1.54, 1.86); in hypertensive and normal populations, the OR values were (1.23, 1.48); in diabetic patients and normoglycemic patients were (1.36,1.43). This proves that it works for all groups of people.

**Summary:**

Our studies demonstrate a strong connection between METS-FV and the emergence of kidney stones. It would be beneficial to investigate METS-VF as a marker for kidney stone development and progression in light of these findings.

## Introduction

An accumulation of crystalline substances, such as uric acid, calcium oxalate, and calcium phosphate, at the point where the renal pelvis and ureter join can lead to the development of kidney stones, a common urinary disorder (1, 2). Various metabolic imbalances (e.g. hyperparathyroidism, hypercortisolism, hyperglycemia), lack of physical activity, lack of essential vitamins and minerals (3)(such as Vitamin B6 and magnesium), blockage of the urinary tract, infection, foreign objects, and drug use are all potential causes of kidney stone formation (4, 5). Though the symptoms of kidney stones may be less visible than those of urolithiasis, they can be more severe and can significantly reduce a patient’s quality of life, with the possibility of developing hematuria, pain, urinary tract infection, and renal dysfunction (6). Consequently, it is essential to acquire a better comprehension of the potential causes that could lead to its emergence.

Having an elevated Body Mass Index (BMI) is the most commonly used way to gauge if one is at risk of forming kidney stones (7, 8), which can be caused by being overweight. However, BMI is not without its drawbacks (9). For example, BMI does not take into account the difference between lean and fat bodies. The METS-VF is an assessment of fat metabolism inside the body which utilizes the insulin resistance index (IR),age, waist-to-height ratio (WHtR), and gender to create a score (10). MRI and BIA have been employed to ascertain the amount of visceral adipose tissue in overseas populations, and it has been proven to be more effective than other surrogate measurements of VAT (11). It can prevent diabetes and fight high blood pressure (12).

We hypothesize that METS-VF could potentially play a role in the emergence and advancement of kidney stones. We conducted a nationwide inquiry to examine the correlation between METS-VF and kidney stones, taking into account numerous potential factors that could affect the possibility of developing kidney stones. The initial inquiry into whether METS-VF is linked to the likelihood of developing kidney stones will begin here.

## Materials and methods

### Study population

**Figure 1:**
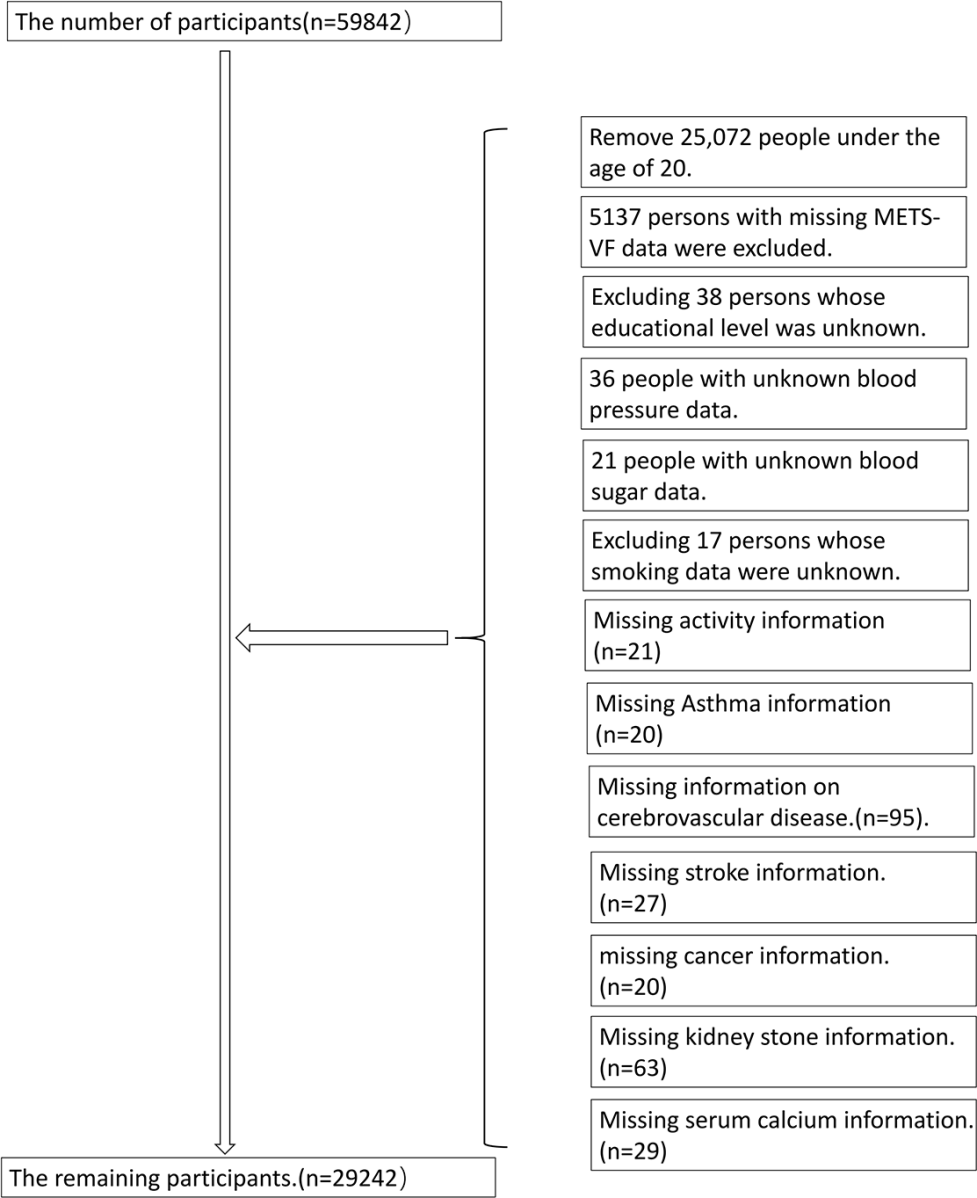
Description of the study population.

This project was carried out in conjunction with the CDC Institutional Review Committee, who utilized complex data analysis methods to analyze the data from the NHANES database; furthermore, all individuals involved gave their consent. Every year, the National Center for Health Statistics conducts a survey that evaluates a certain group of people at a single moment. The National Health and Nutrition Examination Survey is an investigation implemented in the United States to evaluate the wellbeing and dietary habits of a broad range of individuals, particularly those from ethnic minorities and those with low incomes. The research encompassed a total of 29,246 participants (**Figure 1**).

### Data collection and definition

The visceral fat metabolism rating was taken into consideration as a factor in the experiment. We defined METS-VF as 4.466 + 0.011*(Ln(METS-IR))^3 + 3.239*(Ln(WHtr))^3 + 0.319*(Sex) + 0.594*(Ln(Age)).The multivariable models indicated potential outside factors that may interfere with the connection between METS-VF and kidney stones. In this research, sex (male/female), race, educational attainment, marital status (married/single), alcohol intake (drinker/non-drinker), high blood pressure, diabetes, smoking (smoker/non-smoker) and physical activity were all taken into consideration. The exact severity is unknown.

### Statistical methods

The analysis employed an appropriate sample size from NHANES, and a complicated multi-stage cluster survey design was taken into account. The R language’s survey design package was employed to illustrate the intricate multi-stage hierarchical sampling technique of NHANES with the help of the weights from the dataset. To put it succinctly, study-weighted means and 95% confidence intervals are used to illustrate continuous variables, while study-weighted means and 95% confidence levels are applied to describe categorical variables. The linear regression model with study-weighted adjustments was used to determine the impact of the groups on continuous data, and the study-weighted chi-square tests were applied to investigate the effects of the groups on categorical data. The three cohorts were studied to ascertain the relationship between METS-VF and kidney stones *via* the utilization of a multivariate Logistic regression model that was based on pre-defined protocols. Model 1 did not incorporate any changes to the covariates. Model 2 was tailored to take into account characteristics such as gender, ethnicity, educational level, marital status, hypertension, and diabetes. The parameters of Model 3 were modified to include all types of illnesses. In order to gain a more comprehensive insight into the correlation between METS-VF and kidney stones, a penalty spline technique and a generalized additive model regression were employed to fit the data. The saturation threshold effect was tested using the maximum link-like natural ratio to determine the potential threshold value when non-linear relationships exist.

Values with a likelihood lower than 0.05 were considered to be statistically significant. The analyses were conducted using both Empower^®^ software (www.empowerstats.com; X&Y Solutions, Inc., Boston, MA, USA) and R 4.0.2 (http://www.r-project.org, The R Foundation).

## Results

### Increased METS-VF levels are associated with a higher chance of developing kidney stones

**Table 1** outlines the demographics of the participants involved in the study and provides a visual representation of the proportion of characteristics. The results from **Table 1** showed that the stone group had significantly higher METS-VF levels compared to the standard population group. The METS-VF score may be related to a higher rate of kidney stones, and the VIF analysis of the covariates indicated that all VIF values were less than 5, suggesting that there were no issues with collinearity in the data. The logistic regression showed that there was a significant relationship between METS-VF and the development of kidney stones, with Model 3 taking into account all other variables; for each one unit rise in the METS-VF index, there was a 43% higher risk of kidney stones (OR=1.43,95%CI:1.32-1.55). We split METS-VF into three sections and re-ran the logistic regression analysis, which revealed that the top section had a higher value than the bottom part in the third model.

**Table 1:** The characteristics of the participants selected.

Characteristic	Nonstone formers N=26492	Stone formers N=2754
Age(years)	46.56 (46.09,47.02)	53.17 (52.53,53.80)
Cholesterol(MG/DL)	194.11 (193.11,195.10)	192.39 (189.89,194.89)
Serum Calcium (MG/DL)	9.39 (9.38,9.41)	9.37 (9.34,9.40)
Serum Creatinine (MG/DL)	0.87 (0.87,0.88)	0.93 (0.91,0.94)
METS-VF	5.90 (5.88,5.92)	6.02 (5.97,6.07)
Gender(%)		
Male	47.74 (47.04,48.44)	55.46 (52.78,58.11)
Female	52.26 (51.56,52.96)	44.54 (41.89,47.22)
Race(%)		
Mexican American	14.98 (13.09,17.10)	11.30 (9.34,13.61)
White	65.65 (62.78,68.42)	76.97 (73.84,79.83)
Black	11.15 (9.78,12.69)	5.66 (4.67,6.83)
Other Race	8.21 (7.36,9.15)	6.07 (4.87,7.54)
Education Level(%)		
Less than high school	20.39 (18.97,21.89)	19.72 (17.79,21.81)
High school	28.66 (27.48,29.87)	31.44 (28.63,34.39)
More than high school	50.95 (49.08,52.82)	48.84 (45.69,52.00)
Marital Status(%)		
Cohabitation	63.40 (62.12,64.66)	69.45 (66.85,71.93)
Solitude	36.60 (35.34,37.88)	30.55 (28.07,33.15)
Alcohol(%)		
Yes	61.15 (59.67,62.61)	60.07 (56.99,63.07)
No	18.50 (17.44,19.61)	19.27 (17.06,21.69)
Unclear	20.35 (19.25,21.50)	20.66 (17.97,23.64)
High Blood Pressure (%)		
Yes	29.65 (28.65,30.67)	46.24 (43.34,49.17)
No	70.35 (69.33,71.35)	53.76 (50.83,56.66)
Diabetes(%)		
Yes	8.49 (8.02,9.00)	17.49 (15.75,19.37)
No	91.51 (91.00,91.98)	82.51 (80.63,84.25)
Smoked(%)		
Yes	43.57 (42.36,44.79)	49.41 (46.56,52.26)
No	56.43 (55.21,57.64)	50.59 (47.74,53.44)
Physical Activity (%)		
Never	25.99 (25.01,26.99)	29.75 (27.52,32.08)
Moderate	31.97 (30.99,32.96)	31.42 (29.12,33.81)
Vigorous	42.04 (40.93,43.17)	38.83 (36.10,41.63)
Asthma (%)		
No	85.52 (84.82,86.20)	82.75 (80.77,84.57)
Yes	14.48 (13.80,15.18)	17.25 (15.43,19.23)
Coronary Heart Disease (%)		
Yes	3.07 (2.70,3.49)	6.28 (5.20,7.57)
No	96.93 (96.51,97.30)	93.72 (92.43,94.80)
Cancers(%)		
Yes	9.33 (8.85,9.84)	15.77 (14.24,17.43)
No	90.67 (90.16,91.15)	84.23 (82.57,85.76)
PIR(%)		
<1.39	20.13 (18.90,21.43)	18.02 (16.29,19.90)
1.39-3.49	32.50 (31.25,33.77)	35.06 (32.48,37.73)
≥3.49	40.04 (38.16,41.94)	40.05 (36.78,43.42)
Unclear	7.33 (6.69,8.03)	6.86 (5.61,8.37)
Total Kcal (%)		
Tertile 1	39.11 (38.29,39.94)	40.24 (38.01,42.51)
Tertile 2	46.02 (45.04,47.01)	46.53 (43.81,49.26)
Tertile 3	14.87 (14.08,15.69)	13.23 (11.50,15.18)
Total Sugar (%)		
Tertile 1	36.46 (35.61,37.33)	36.73 (33.94,39.63)
Tertile 2	37.22 (36.2,38.19)	37.09 (34.15,40.14)
Tertile 3	26.31 (25.50,27.14)	26.17 (23.87,28.61)
Total Water (%)		
Tertile 1	38.92 (38.00,39.84)	37.39 (34.92,39.94)
Tertile 2	46.22 (45.28,47.16)	49.38 (46.60,52.15)
Tertile 3	14.87 (14.08,15.69)	13.23 (11.50,15.18)
Drxttfat.D2.New (%)		
Tertile 1	38.92 (38.00,39.84)	37.39 (34.92,39.94)
Tertile 2	46.22 (45.28,47.16)	49.38 (46.60,52.15)
Tertile 3	14.87 (14.08,15.69)	13.23 (11.5,15.18)

### Subgroup analysis

We also controlled for sex, race, hypertension, and diabetes using subgroup analysis. The univariate logistic regression between kidney stone prevalence and METS-VF showed a positive relationship, with an odds ratio of 1.49 (95%CI:1.33-1.67) compared to 1.44 (95%CI: 1.29 – 1.62), (**Table 2**). The likelihood of Mexicans being affected by the outcome was 1.33 times greater than the general population (95% CI 1.11-1.60). The average odds ratios for whites, blacks and other races (non-Mexican) were respectively 1.28-1.59 (95% confidence interval), 1.25-1.89 (95% confidence interval), and 1.42-2.44 (95% confidence interval). The hypertensive group had an odds ratio of 1.23 (95% CI 1.08 to 1.41), while the nonhypertensive group had an odds ratio of 1.48 (95% CI 1.34 to 1.64) in the hypertension stratification. When segregated by diabetes, the probability ratio was 1.36 (95% CI 1.07–1.72) for those with diabetes and 1.43 (95% CI 1.32–1.56) for those without.

**Table 2:** Subgroup analysis between METS-IR index with kidney stone formation.

Exposure	Model1	Model2	Model3
METS-VF	1.07 (1.03,1.11)	1.81 (1.68,1.94)	1.43 (1.32,1.55)
Gender (%)			
Male	1.73 (1.58,1.91)	1.92 (1.72,2.13)	1.49 (1.33,1.67)
Female	1.76 (1.60,1.95)	1.78 (1.60,1.97)	1.44 (1.29,1.62)
Race (%)			
Mexican American	1.01 (0.94,1.08)	1.84 (1.55,2.18)	1.33 (1.11,1.60)
White	1.05 (1.00,1.11)	1.77 (1.60,1.95)	1.43 (1.28,1.59)
Black	1.23 (1.11,1.36)	1.89 (1.56,2.28)	1.54 (1.25,1.89)
Other Race	1.36 (1.16,1.58)	2.43 (1.89,3.12)	1.86 (1.42,2.44)
High Blood Pressure (%)			
Yes	0.91 (0.86,0.96)	1.38 (1.21,1.57)	1.23 (1.08,1.41)
No	1.07 (1.02,1.13)	1.67 (1.52,1.84)	1.48 (1.34,1.64)
Diabetes (%)			
Yes	0.87 (0.80,0.95)	1.46 (1.16,1.84)	1.36 (1.07,1.72)
No	1.07 (1.03,1.12)	1.67 (1.54,1.81)	1.43 (1.32,1.56)

Model 1 = no covariates were adjusted.

Model 2 = Model 1+ GENDR, RACE,EDUCATION, MARITAL.

Model 3 = adjusted for all covariates except effect modifier.

*Means only in model 3.

### Dose-response and threshold effect analysis of the METS-VF index on the prevalence of kidney stones

**Figure 2:**
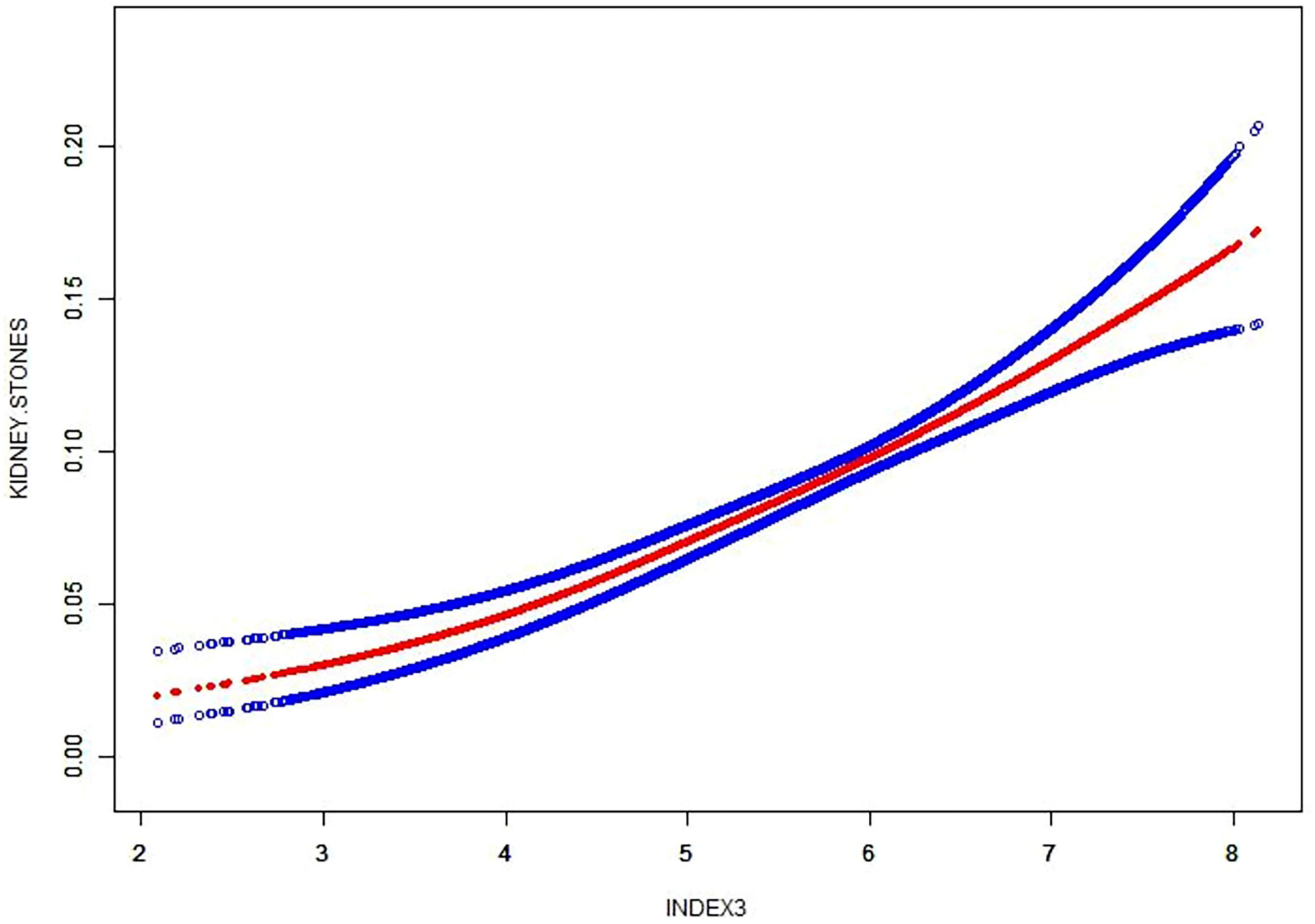
Density dose–response relationship between METS-VF with kidney stone formation. The area between two dotted lined is expressed as a 95% CI.

A research project was conducted to investigate the link between the METS-VF index and the frequency of kidney stones, utilizing a generalized additive model and a smooth curve fitting approach. Our study has revealed a significant link between the METS-VF index and the occurrence of kidney stones (**Figure 2**).

## Discussion

Our investigation has revealed a significant association between METS-VF and the development of kidney stones. After accounting for potential confounding variables, this relationship remained significant. Our findings provide valuable insight into the etiology of nephrolithiasis and suggest the need for further research to explore the underlying mechanisms of this association.METS-VF is an accurate measure for assessing the likelihood of kidney stones and can be utilized to monitor their progression. The index measures total energy expenditure during physical activity, as well as the frequency and intensity of activity. Furthermore, this index has been found to be a useful tool for monitoring kidney stone progression in those who already have kidney stones. This is the initial assessment to ascertain if there is a relationship between METS-VF and the development of kidney stones. More in-depth study should be conducted to gain a clearer comprehension of the correlation between METS-VF and kidney stones, as well as to pinpoint other possible sources of risk. This research could pave the way for more successful approaches to averting and managing kidney stones.

Carrying excess weight around the abdomen increases the likelihood of developing kidney disease (13). Adipose tissue helps maintain the equilibrium between food intake and energy expenditure through the production of adipokines under normal physiological conditions. The adipose tissue contributes significantly to the maintenance of the nutritional balance and energy metabolism of the body by releasing a variety of molecules called adipokines, which can influence appetite, metabolism, inflammation, and other bodily functions. These adipokines are secreted in response to various cues, such as nutrient availability, hormones, and physical activity, allowing the body to effectively adjust energy and nutrient levels. The importance of adipose tissue in controlling these activities is paramount for achieving optimal health and well-being. When abnormalities occur, the overgrowth and build-up of fatty tissue can cause biological abnormalities and dysfunction, which can result in the deposition of excessive fat in other areas and eventually lead to malfunctioning organs. Pathological conditions that lead to an overgrowth and buildup of fat cells can result in biological abnormalities and disruption of the fatty tissue. Pathological states that cause an abnormal increase in fat deposits can lead to a range of biological issues and interfere with the typical functioning of fatty tissue. This can negatively affect a person’s health and well-being and can lead to further complications. It is important to monitor fatty tissue health and take steps to maintain healthy body composition, to avoid these adverse effects. Associated with this is the accumulation of ectopic fat, which can lead to impaired organ function. The accumulation of ectopic fat is a serious medical condition that can have far-reaching consequences. It occurs when fat accumulates in areas other than the body’s normal fat deposits, such as the liver, heart, and muscles. This can lead to impaired organ function, including heart and liver damage, insulin resistance, and even an increased risk of certain types of cancer. Therefore, it is important to understand the potential risks associated with ectopic fat accumulation and take steps to reduce the risks. This includes maintaining a healthy diet and exercising regularly. It is also important to talk to your doctor if you are at risk for this condition. This can have serious consequences for a person’s health and well-being. The repercussions of poor health and well-being can be far-reaching and long-lasting. From physical and mental health issues to financial and social difficulties, the effects can be devastating. When individuals do not prioritize their health and well-being, it can cause a ripple effect in all aspects of life. It can cause a drop in efficiency on the job, a lack of attention and focus, and an overall reduction in well-being. It is essential to prioritize and maintain health and well-being, as it can have serious implications for an individual’s life. It is critical to be mindful of the potential issues that can stem from adipose tissue disorders, and to reach out to a medical professional if any warning signs occur. It is critical to be mindful of the risks of pathological adipose tissue and to take proactive steps to address any signs of dysfunction. Adipose tissue dysfunction can manifest in a variety of ways, including weight gain, excessive fat deposition, and metabolic disturbances. If any of these symptoms or signs occur, seek medical advice from a qualified healthcare practitioner so that the situation can be assessed and an appropriate treatment plan can be instituted. Early detection and treatment of adipose tissue dysfunction can help prevent further complications and may even help to reverse existing conditions. Multiple investigations have been conducted to explore the relationship between abdominal fat and kidney performance. A research paper examining the relationship between obesity, metabolic disorders, and CKD progression revealed that individuals with obesity and metabolic disorders had an elevated risk of CKD compared to those without such conditions (14). This research is comparable to our study in that they both explore the association between fat and kidney damage, however, it is a broad analysis of the relationship between obesity and kidney injury and not a more nuanced examination of the different kinds of fat, whereas our study is a more in-depth look at the link between visceral fat accumulation and kidney stones, with more convincing results. Kang and their associates used bioelectrical impedance measurements at different frequencies to determine visceral fat levels and observed a clear association between increased amounts of visceral fat and an amplified risk of chronic kidney damage. This link persisted even when accounting for factors like age, gender, diabetes, and high blood pressure (15). Instead of using the multifrequency bioelectric impedance analysis method to assess visceral body fat, we opted for METS-VF which made our research more feasible and enabled us to include a greater number of participants. Additionally, visceral fat has been linked to the progression of kidney disease. In Japan, researchers utilized computed tomography to evaluate the proportion of visceral and subcutaneous fat in patients with preexisting chronic kidney disease. They found that individuals with a higher ratio of visceral to subcutaneous fat experienced a reduction in eGFR of at least 30% (16).

This research serves as a starting point for further study of the association between METS-FV and kidney stones, thus emphasizing the relevance of METS-VF in the creation of kidney stones. This study has shown that the collaboration between these two elements is essential for this condition. This extensive research showed that individuals who engaged in higher levels of strenuous physical activity were more likely to develop kidney stones. The significance of regular physical activity in keeping kidney health in check is underscored by this discovery. More studies must be conducted to gain an in-depth knowledge of the precise processes that connect physical activity to the development of kidney stones.This research proposes that METS-VF, a marker of visceral fat, could possibly be used to anticipate the appearance and advancement of kidney stones. The rise of visceral fat has been linked to the start of metabolic syndrome and type 2 diabetes, which both raise the odds of kidney stones forming. Moreover, elevated quantities of visceral fat can cause heightened levels of particular hormones and inflammatory substances, which can be responsible for the emergence and worsening of kidney stones. Consequently, additional investigations should be conducted to assess if METS-VF can be utilized as a reliable marker of kidney stone development and advancement. The results of this study indicated that the relationship studied had strong implications when broken down by sex, ethnicity, hypertension, and diabetes, implying that it is relevant across all populations. The researchers analyzed the association between two factors, and broke down the results by gender, ethnicity, hypertension, and diabetes. The results of our study suggested that this relationship had distinct impact levels when grouped by gender, ethnicity, hypertension, and diabetes, implying that it could be pertinent to a broad range of individuals. The possible reasons are as follows: METS-VF is an indicator of obesity (17), Obese people usually have higher fat and calorie intake and control high blood pressure and diabetes (18, 19), Thus reducing the correlation of METS-VF with kidney stones compared to the disease.

We have several hypotheses regarding the relationship between visceral fat and kidney stones, including: ① An overabundance of fat which causes triglyceride droplets to accumulate in the tubular epithelial cells, high cholesterol levels in the podocytes that disrupt the wall’s stability, and high glucose levels stimulating fat gene expression and reducing β-oxidation. The abnormal glucose and lipid metabolism contribute to a disruption of cellular structure and inflammation of the kidneys, resulting in the formation of kidney stones. ②A rise in visceral fat may be associated with an elevation in serum uric acid levels. This heightens the likelihood of uric acid stones forming in the kidneys (20). ③Excess body weight can cause an increase in the amount of sodium that is reabsorbed by the kidneys, thus activating the renin-angiotensin and sympathetic nervous systems (21), which in turn leads to an increased prevalence of nephrolithiasis (22). ④In obese patients, the fat around the kidney can compress the kidney, leading to intraarterial hypertension around the kidney and then nephrogenic hypertension (23). It is widely accepted that having elevated blood pressure can significantly increase the likelihood of developing kidney stones.

This study offers a thorough examination of the correlation between METS-VF and the potential for kidney stone formation, an area that has seen relatively little prior investigation. This research is groundbreaking in that it marks the first investigation of the association between METS-VF and kidney stone development (24). This study provides crucial information on the possible link between the two conditions, as well as the potential consequences for diagnosing and treating kidney stones. In addition, this research may pave the way for further investigation into the correlation between METS-VF and kidney stone prevalence, potentially leading to better patient care (25). Despite this, our research has four significant drawbacks. To begin with, we took into account factors such as ethnicity, gender, educational attainment, matrimonial status, exercise routine, smoking habits, alcohol intake, blood pressure, and blood glucose levels to control for any confounding effects. It is possible that other yet-unidentified or unmeasured elements could impact the accuracy of METS-VF in predicting the likelihood of kidney stones. Additionally, it is difficult to accurately measure visceral fat with MRI or CT scans due to a lack of reliable data. Finally, because the number of participants in our research was limited, it is not clear if METS-VF can be used to anticipate the occurrence of kidney stones in other groups. Fourth, a cross-sectional study found no specific causal relationship between METS-VF and kidney stone prevalence.

## Conclusion

Our research, taking into account a plethora of US population-related data, revealed an incredibly strong correlation between visceral fat metabolism index and the emergence and progression of kidney stones. Our research could offer novel approaches for the avoidance and management of kidney stones. Further exploration is essential to uncover the underlying causes of this phenomenon.

## Data availability statement

The original contributions presented in the study are included in the article/supplementary material. Further inquiries can be directed to the corresponding authors.

## Author contributions

ZG, GL, YC: Conceptualization, Methodology, Software. SS, SF: Visualization, Investigation. WW, YH: Writing - review and editing. All authors contributed to the article and approved the submitted version.
