# Corrigendum: Could METS-VF provide a clue as to the formation of kidney stones?

**DOI:** 10.3389/fendo.2023.1357653

**Published:** 2024-01-15

**Authors:** Zhenyu Guo, Guoxiang Li, Yan Chen, Shuai Fan, Shuai Sun, Yunwu Hao, Wei Wang

**Affiliations:** ^1^ Department of Urology, the First Affiliated Hospital of Anhui Medical University, Hefei, China; ^2^ Institute of Urology, Anhui Medical University, Hefei, China; ^3^ Anhui Province Key Laboratory of Genitourinary Diseases, Anhui Medical University, Hefei, China; ^4^ Department of General Practice, Wuhu City Second People`s Hospital, Wuhu, China; ^5^ Department of Urology, Lu’an Hospital Affiliated of Anhui Medical University, Lu’an, China

**Keywords:** METS-VF, kidney stones, obesity, NHANES, clue

**Missing author contribution symbol.**

In the published article, the equal contribution symbol was mistakenly not included alongside author Yan Chen’s name. This has now been added.

The authors apologize for this error and state that this does not change the scientific conclusions of the article in any way. The original article has been updated.

